# Localization and local translation of *Arc*/*Arg3.1* mRNA at synapses: some observations and paradoxes

**DOI:** 10.3389/fnmol.2014.00101

**Published:** 2015-01-12

**Authors:** Oswald Steward, Shannon Farris, Patricia S. Pirbhoy, Jennifer Darnell, Sarah J. Van Driesche

**Affiliations:** ^1^Reeve-Irvine Research Center, University of California IrvineIrvine, CA, USA; ^2^Department of Anatomy and Neurobiology, University of California IrvineIrvine, CA, USA; ^3^Department of Neurobiology and Behavior, University of California IrvineIrvine, CA, USA; ^4^Center for the Neurobiology of Learning and Memory, University of California IrvineIrvine, CA, USA; ^5^Laboratory of Molecular Neuro-Oncology, The Rockefeller UniversityNew York, NY, USA

**Keywords:** LTP, synaptic plasticity, protein synthesis, dendrite, dendritic mRNA, dendritic spines, immediate early genes, *Arc*/*Arg3.1*

## Abstract

*Arc* is a unique immediate early gene whose expression is induced as synapses are being modified during learning. The uniqueness comes from the fact that newly synthesized *Arc* mRNA is rapidly transported throughout dendrites where it localizes near synapses that were recently activated. Here, we summarize aspects of *Arc* mRNA translation in dendrites *in vivo*, focusing especially on features of its expression that are paradoxical or that donot fit in with current models of how Arc protein operates. Findings from *in vivo* studies that donot quite fit include: (1) Following induction of LTP *in vivo*, *Arc* mRNA and protein localize near active synapses, but are also distributed throughout dendrites. In contrast, *Arc* mRNA localizes selectively near active synapses when stimulation is continued as *Arc* mRNA is transported into dendrites; (2) Strong induction of *Arc* expression as a result of a seizure does not lead to a rundown of synaptic efficacy *in vivo* as would be predicted by the hypothesis that high levels of Arc cause glutamate receptor endocytosis and LTD. (3) *Arc* protein is synthesized in the perinuclear cytoplasm rapidly after transcriptional activation, indicating that at least a pool of *Arc* mRNA is not translationally repressed to allow for dendritic delivery; (4) Increases in *Arc* mRNA in dendrites are not paralleled by increases in levels of exon junction complex (EJC) proteins. These results of studies of mRNA trafficking in neurons *in vivo* provide a new perspective on the possible roles of Arc in activity-dependent synaptic modifications.

In response to experience, Arc

Travels out to the dendrites to park

We would all love to see

What its functions might be

But we’re pretty much still in the dark

Limerick by anonymous University of Illinois neuroscientist, continuing the tradition of William T. Greenough.

## INTRODUCTION

It is widely believed that memory storage involves modifications of synaptic properties that are induced by specific patterns of activity, and that enduring forms of plasticity require gene expression and mRNA translation (Kandel, 2001; Dudai, 2002; Martin and Morris, 2002). Some forms of synaptic modifications appear to require protein synthesis as the modifications are induced (Pfeiffer and Huber, 2006); other forms can be induced when protein synthesis is blocked, but are not maintained without protein synthesis (Kandel, 2001). A useful term to refer to the process through which initially transient and labile changes are rendered more permanent is “synaptic consolidation.” This term recognizes the formal similarity to the process of memory consolidation, through which labile memories become more resistant to disruption over time. Although formally similar, there is no compelling evidence that the similarities between synaptic consolidation and memory consolidation indicate shared mechanisms.

Activity-dependent synaptic modifications that require protein synthesis at the time of induction presumably involve translation of mRNAs that are constitutively present. On the other hand, synaptic modifications that require new gene expression must involve signaling from active synapses to the nucleus to induce gene transcription and then delivery of newly synthesized gene products back to the synapses that are to be modified. Identifying the genes that are critical for synaptic modifications and defining how the gene products act has been a long-standing goal of modern neuroscience. In this regard, mechanisms that could allow activity-dependent alterations of individual synapses or small clusters of synapses in a way that involves gene transcription and mRNA translation are of particular interest (Steward and Schuman, 2003; Hirokawa, 2006; Schuman et al., 2006).

One potential mechanism that would allow activity-dependent modification of individual synapses has been revealed through studies of the unique immediate early gene (IEG) called *Arc* (activity-regulated cytoskeleton associated protein; Lyford et al., 1995), also known as *Arg3.1* (Link et al., 1995). For simplicity, we will use the term *Arc* hereafter. *Arc* has become a model for studies of mRNA trafficking because of several very unique features. Like other IEGs, *Arc* transcription is strongly induced by synaptic activity (Steward and Worley, 2001a) and behavior (Guzowski et al., 1999). *Arc* mRNA is unique amongst IEGs, however, because the newly synthesized mRNA transcript is rapidly transported into dendrites (Link et al., 1995; Lyford et al., 1995; Wallace et al., 1998). Newly synthesized *Arc* mRNA localizes in a highly selective fashion near synapses that have recently experienced patterns of activity sufficient to activate NMDA receptors (Steward et al., 1998; Steward and Worley, 2001b). Arc protein associates with the post-synaptic density, and elegant studies indicate that Arc plays a role in AMPA receptor endocytosis, thereby contributing to down-regulation of synaptic efficacy at excitatory synapses (Chowdhury et al., 2006; Rial Verde et al., 2006; Shepherd et al., 2006). The induction, delivery of *Arc* mRNA to dendritic domains contacted by active synapses, and local synthesis of Arc protein thus provides a model that explains how individual synapses could be modified in an activity-dependent and gene expression-dependent manner (Steward and Worley, 2001a). This mechanism is of even more interest because of evidence that antisense-mediated abrogation of Arc protein synthesis disrupts memory consolidation (Guzowski et al., 2000).

Here, we review some of the key data documenting features of *Arc* expression and mRNA localization at active synapses. Several recent reviews have focused on the many ways that Arc’s characteristics meet expectations for a molecule that is critically involved in synaptic modifications underlying memory consolidation (Steward et al., 2014). Here, we consider the other side of the story, that is, some of the details about Arc that are unexpected based on proposed mechanisms or that do not quite fit the story.

## MATERIALS AND METHODS

### ELECTROPHYSIOLOGY TECHNIQUES

Experiments were carried out using adult, male and female Sprague Dawley rats. Rats were anesthetized via intraperitoneal injections of 20% urethane (500 mg/kg body weight) given approximately every 10 min until the animal was totally unresponsive to tail pinch. Rats were positioned in a stereotaxic apparatus and burr holes were placed in the skull to allow placement of stimulating and recording electrodes. An insulated monopolar stimulating electrode was positioned stereotaxically at 4.0 mm lateral to the midline and 1.0 mm anterior to the transverse sinus. The depth of the stimulating electrode was adjusted so as to maximally activate the medial perforant path (MPP) originating from the medial entorhinal cortex (EC) – usually 3–4 mm below the cortical surface. Glass recording electrodes filled with 0.9% saline were positioned at 1.5–2.0 mm lateral to the midline, and 3.5 mm posterior to bregma. Electrodes were positioned in the dorsal blade of the dentate gyrus (DG) so as to record field potentials from the cell body layer.

### STIMULATION PARADIGM

After positioning the stimulating and recording electrodes, stimulus intensity was set so as to evoke a population spike of ∼3–6 mV. Single test pulses were delivered at a rate of 1/10 s at the same intensity for 10 min in order to determine baseline response amplitude, measuring the slope of the population excitatory postsynaptic potential (EPSP) and amplitude of the population spike. Following baseline recordings, three rounds of high frequency stimulation (HFS) were given, with each round consisting of ten trains of eight pulses at 400 Hz and each train given at a rate of 1/10 s. After each bout of HFS, a round of ten test pulses was given to determine the extent of potentiation of synaptic responses. After the third round of test pulses, either test stimulation or HFS was continued as described in the Results.

### ELECTROCONVULSIVE SEIZURES

A single electroconvulsive seizure (ECS) was induced in young adult Sprague Dawley rats as described previously (Wallace et al., 1998). Current was passed transcranially (40 mA for 0.5 s) via ear clip electrodes resulting in a generalized tonic/clonic seizure that lasted ∼15 s.

### TISSUE PREPARATION

Rats were killed by a lethal injection of the anesthetic Euthasol or sodium pentobarbital 100 mg/kg depending on the IACUC protocol in effect on the date of the experiment. For immunocytochemistry and non-isotopic *in situ* hybridization, rats were perfused with 4% paraformaldehyde in phosphate buffered saline (PBS, pH 7.4). Brains were sectioned on a Vibratome® at 40 μm and stored in 1x PBS at 4°C with sodium azide. For fluorescence *in situ* hybridization (FISH), rats were killed by decapitation, brains were removed and rapidly frozen, and brains were sectioned on a cryostat (for additional details, see Farris et al., 2014).

### IMMUNOCYTOCHEMISTRY

For Arc immunocytochemistry, free-floating Vibratome sections were heated to 95°C for 5 min for antigen retrieval and treated with H_2_O_2_ to block endogenous peroxidase. Sections were then blocked in 10% natural goat serum and 0.1% Triton in PBS for 1 h at RT before overnight incubation with a mouse monoclonal Arc antibody for 18 h (Santa Cruz Biotechnology, SC-17839, 1:100 dilution). The sections were washed in PBS, incubated for 2 h with biotinylated goat anti-mouse IgG secondary antibody (Vector Laboratories, BA-9200, 1:250 dilution), then incubated for 1 h in Vectastain® ABC kit (Vector Laboratories, PK-6100) for horseradish peroxidase (HRP) deposition. The HRP was detected using a signal amplification system, tyramide-FITC in 0.1 M borate buffer pH 8.5 with 0.003% H_2_O_2_ for 30 min (Invitrogen, C1311, 1:250 dilution). Sections were then washed with PBS, mounted and coverslipped using Vectashield® (Vector Laboratories). For immunostaining with antibodies against RNA binding proteins (RBPs), sections were washed in PBS and blocked with TSA blocking buffer (Perkin Elmer) before overnight incubation with the following antibodies generated in rabbit; anti-Staufen2, anti-Barentsz (both generous gifts from Dr. Michael Kiebler 1:200), anti-Upf1 (generous gift from Dr. Jens Lykke-Andersen 1:200), or anti-eIF4A111 (generous gift from Dr. Nahum Sonenberg 1:200). The tissue was washed in PBS and then was incubated with goat-anti rabbit-HRP (Jackson Immunoresearch 1:250) followed by Cy3-Tyramide amplification (Perkin Elmer) according to the manufacturer’s protocol.

### *IN SITU* HYBRIDIZATION

The cRNA probe for *Arc*/*Arg3.1* has been described previously (Steward et al., 1998). For non-radioactive *in situ* hybridization (NRISH) slide-mounted sections or floating vibratome sections were post-fixed with 4% paraformaldehyde in 0.1 M PBS for 30 min, then rinsed with 0.5x saline-sodium citrate buffer (0.5x SSC, 0.1% DEPC treated) for 5 min. Sections were treated with Proteinase K (1.25 mg/L) for 30 min, rinsed again with 0.5x SSC (0.1% DEPC treated) for 10 min and air-dried. The sections were covered with 75 μl prehybridization buffer (2x SSC, 25% formamide, 1% Denhardt’s reagent, 10% dextran sulfate, 0.5 mg/mL heparin, 0.5 mg/ml yeast tRNA, and 0.25 mg/mL of denatured salmon sperm DNA) and incubated at 42°C for 2 h. After the prehybridization, about 0.5 μg of Dig-cRNA probe in 75 μl hybridization buffer was added to each section. The sections were covered with a baked coverslip, and incubated overnight at 55°C in a humidified box with 25% formamide/2x SSC. The next day, the coverslips were removed and sections were washed with 2x SSC/10 mM EDTA twice (10 min each). The sections were treated with RNAse-A for 30 min, and then washed twice with 2x SSC/EDTA (10 min per wash). The stringency wash was 0.5x SSC/10 mM EDTA at 55°C for 2 h. Sections were washed with 0.5x SSC twice (10 min each at room temperature). Alkaline phosphatase conjugated anti-digoxigenin Fab fragment (1:5000) was used to detect the hybridized probes. NBT/BCIP solution was applied overnight at 4°C to detect the alkaline phosphatase. Sections were washed with 100 mM Tris-HCl (pH 8.5)/1 mM EDTA three times, 10 min each. Then slides were briefly rinsed with nanopure water twice and covered with Kaiser’s glycerol jelly.

Fluorescent *in situ* hybridization for *Arc*/*Arg3.1* RNA was performed on 20 μm coronal sections prepared from flash-frozen brains as described by Guzowski et al. (1999). The *Arc*/*Arg3.1* cRNA riboprobe was generated using the Ambion MaxiScript kit and a premixed RNA labeling nucleotide mix containing digoxigenin-labeled UTP (Roche Molecular Biochemicals). Brain sections were incubated with the digoxigenin-labeled *Arc* antisense riboprobe (1–2 ng/ml) for 16–20 h. Subsequent to washes of various stringencies, the brain sections were incubated with a HRP-conjugated antibody to digoxigenin. The HRP was detected using the Tyramide Signal Amplification fluorescence (TSA-CY3) kit from Perkin Elmer. Finally, nuclei were stained with DAPI and the slides were coverslipped using Vectashield® mounting media (Vector Laboratories).

### POLYRIBOSOME ANALYSIS ON SUCROSE GRADIENTS

Purification of mRNP, monosome and polyribosome fractions by sucrose density centrifugation on linear 20–50% gradients was performed as previously described (Darnell et al., 2005, 2009). Briefly, two FVB adult male littermate mice received an intraperitoneal injection of scopolamine, 1 mg/kg, and a second injection of saline after 30 min. 30 min later the mice were sacrificed by brief exposure to isoflurane and decapitation. The cortex, hippocampus and cerebellum were homogenized in 1ml gradient buffer (20 mM HEPES, 150 mM KCl, 5 mM MgCl2), in the presence of cycloheximide (CHX) plus 1% NP-40. Mitochondria were pelleted by centrifugation and a post-mitochondrial supernatant was applied to 20–50% sucrose gradients prepared in gradient buffer, centrifuged for 2 h at 41,000 rpm in a SW41 rotor and fractionated on an ISCO fractionator with UV detection at 254 nm. RNA was prepared from each of 16 fractions and quantitative RT-PCR for *Arc* mRNA or *Bdnf* mRNA was performed as described below.

### RNA RECOVERY FROM SUCROSE FRACTIONS

*In vitro* transcribed luciferase RNA was spiked into each gradient fraction before RNA preparation to control for RNA recovery. 400 μl of each fraction was mixed with 1.2 ml Trizol-LS (Invitrogen) and RNA purified according to manufacturer’s instructions.

### QUANTITATIVE RT-PCR

Following treatment with RQ1 DNAse (Promega), purified RNA was reverse transcribed using random hexamers (Roche) and Superscript III reverse transcriptase (Invitrogen) according to manufacturer’s protocols. cDNA products were amplified using iTaq SYBR green Supermix with Rox (BioRad) with 200 nM of the following primers.

*Arc F* (spanning exons 2 to 3) 5′-GAGAGCTGAAAGGGTTGCAC-3′
*Arc R* (spanning exons 2 to 3) 5′-GCCTTGATGGACTTCTTCCA-3′
*Bdnf F* (spanning exons 10 to 11) 5′-TGGCTGACACTTTTGAGCAC-3′
*Bdnf R* (spanning exons 10 to 11) 5′-CAAAGGCACTTGACTGCTGA-3′
*Luc* F 5′-GCCTTGATTGACAAGGATGGA-3′
*Luc* R 5′-CAGAGACTTCAGGCGGTCAAC-3′

Quantitative PCR amplification was performed using a BioRad iQ5 real-time RT-PCR detection system. For polyribosome distribution, the relative *Arc* or *Bdnf* mRNA level in each fraction was calculated according to the formula 2^(40-Ct)^ and normalized to luciferase levels calculated in the same way. The amount of mRNA in each fraction was then plotted as a percentage of total mRNA summed over the entire polyribosome gradient. Error bars reflect the technical replicates from triplicate wells in a single representative experiment from polysome gradients. Error is calculated using the formula suggested by the ABI user bulletin, [(std dev for *Fmr1*)^2^ + (std dev for luc)^2^]^0.5^.

## RESULTS

### SELECTIVE LOCALIZATION OF *Arc* mRNA AT ACTIVE SYNAPSES

**FIGURE 1:**
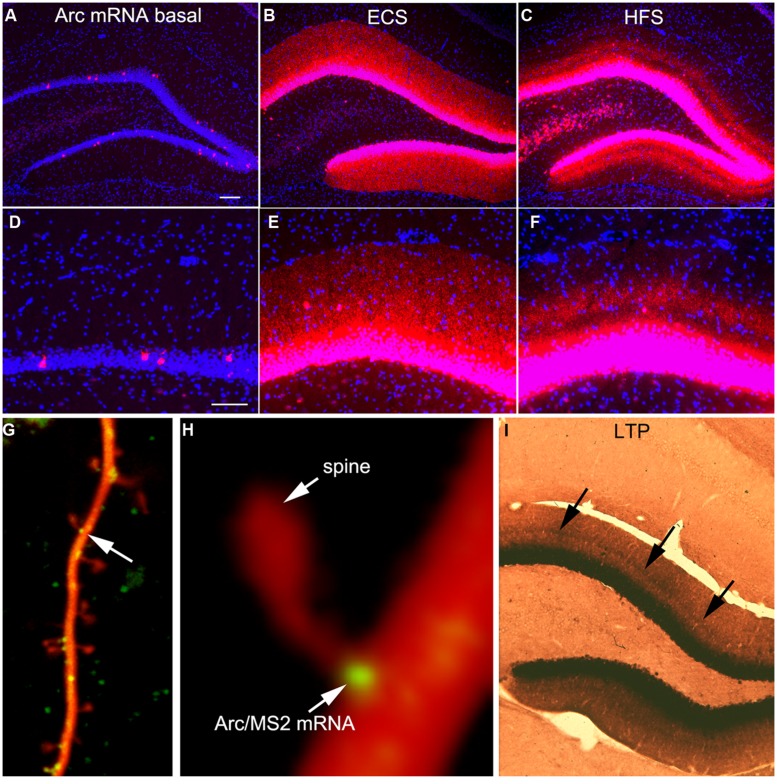
**Activity-dependent induction and *Arc* mRNA and localization near active synapses as revealed by fluorescent *in situ* hybridization (FISH). (A)** *Arc* mRNA is expressed at low levels under basal conditions. The image illustrates the distribution of *Arc* mRNA (red) on the non-stimulated side of an anesthetized rat. **(B)** Induction of *Arc* expression after an electroconvulsive seizure. The rat was euthanized 2 h after the ECS. Note: that *Arc* is expressed by large numbers of dentate granule cells and *Arc* mRNA is transported throughout dendrites. **(C)** Selective targeting of *Arc* mRNA to active synapses. The panel illustrates the selective localization of *Arc* mRNA in the middle molecular layer of the dentate gyrus (DG) after high frequency stimulation of the MPP. **(D–F)** are high magnification views of the fields shown in **(A–C)** respectively. Selective targeting of *Arc* mRNA to the activated portions of dendrites was initially described by Steward et al. (1998). **(G)** Image of a dendrite from a neuron in culture in which immobile *Arc*/MS2 mRNA particles are identified by image averaging over time. **(H)** Higher power view of an *Arc*/MS2 particle parked at the base of a dendritic spine (spine is indicated by the arrow in **G**). The image was smoothed by Gaussian blurring function in Photoshop. **(G,H)** Are from Dynes and Steward (2012). **(I)** Non-isotopic *in situ* hybridization preparation illustrating the distribution of *Arc* mRNA 2 h after inducing LTP with 20 high frequency trains (a minimal stimulation paradigm). Arrows indicate band of increased mRNA levels in the middle molecular layer, but note also that *Arc* mRNA is distributed throughout the dendrites of dentate granule cells.

*Arc* is expressed as an IEG so transcription is strongly induced by synaptic activity. In control rats, *Arc* is expressed at low levels overall; for example, FISH reveals low levels of expression in the hippocampus except for a few neurons scattered in the granule cell layer of the DG (**Figures 1A,D**). Intense neuronal activity (an ECS for example) strongly induces *Arc* transcription and in the absence of other stimulation, newly synthesized *Arc* mRNA is transported throughout dendrites (**Figures 1B,E**). Arc protein expression following an ECS mirrors that of the mRNA; high levels of protein are present throughout the somata and dendrites of dentate granule cells for hours (Farris et al., 2014). As an aside, in all of our studies, Arc protein distribution mirrors the distribution of *Arc* mRNA. This, and other evidence, is the basis of our conclusion that the local levels of Arc protein in different cellular compartments are controlled by the local transcription of *Arc* mRNA (Farris et al., 2014). The only exception to this generalization is the fact that there are high levels of Arc protein in the nucleus, which must reflect nucleocytoplasmic transport following synthesis in the cytoplasm.

One of the most striking properties of Arc is that *Arc* mRNA localizes selectively near active synapses. We documented targeting to active synapses using paradigms in which HFS was continuously delivered to the perforant path at a rate of 1/10 s for 1–2 h (Steward et al., 1998). Synapses of the MPP terminate selectively in a discrete layer on mid proximo-distal dendrites of granule cells. HFS strongly induces *Arc* transcription and newly synthesized *Arc* mRNA is transported into dendrites where it accumulates selectively in the dendritic domains that are contacted by the active synapses (the middle molecular layer, **Figures 1C,F**). Arc protein accumulates in the same lamina as *Arc* mRNA via local synthesis (Steward et al., 1998). Importantly, with perforant path stimulation, *Arc* mRNA does not reach the outer molecular layer, which contains the distal dendrites of dentate granule cells, indicating that when there is ongoing synaptic activation, newly synthesized *Arc* mRNA in transit is captured in activated dendritic segments.

It is important to note that selective localization in the activated portion of dendrites does not necessarily indicate targeting to individual active synapses. Nevertheless, other studies document that mRNA transcripts containing the 3′UTR of *Arc* mRNA localize with remarkable precision in a small domain at the base of dendritic spines (Dynes and Steward, 2012). Neurons in culture were transfected with two DNA constructs. One contains the 3′UTR of *Arc* mRNA, six copies of a sequence that is recognized by the MS2 phage coat protein (the MS2 binding domain), and the coding sequence for red fluorescent protein (DsRE), all of which was expressed under the control of the CMV promoter (CMV-DsRE-6xBS-rArc3′UTR construct). The second construct encoded a fusion protein made up of GFP, MS2 protein, and a nuclear localization sequence (GFP-MS2-NLS construct). When the two constructs are co-expressed, GFP/MS2 fusion protein binds the mRNA with the MS2 domain and is carried with the mRNA out into dendrites. Unbound GFP/MS2 is imported into the nucleus. Using this system, we first showed that that *Arc*/MS2 was transported at a variety of rates up to 70 μm/min. At this rate, newly synthesized *Arc* mRNA could travel from the nucleus to synapses on far distal dendrites in a matter of minutes (Dynes and Steward, 2007). Subsequent studies revealed that *Arc*/MS2 mRNA localized with remarkable precision at the base of dendritic spines (**Figures 1G,H**). Localization persists in the presence of translation inhibitors indicating that localization does not require ongoing translation. Thus, the 3′UTR of *Arc* mRNA is sufficient for localization even when the construct encodes something other than Arc protein, in this case, DsRE (Dynes and Steward, 2012). Again, these results do not demonstrate selective localization at active synapses, but do indicate that mechanisms exist to cause *Arc* mRNA to localize selectively at individual synapses. An intriguing observation from live imaging was that *Arc* mRNA particles at spine bases exhibited highly constrained submicron movements, suggestive of movement of the mRNA during local translation (Dynes and Steward, 2012).

### A PARADOX IN TERMS OF TIMING

The highly selective localization of *Arc* mRNA near active synapses would provide an ideal mechanism for *Arc* transcription to be induced by activation of a particular set of synapses, and then have the mRNA selectively targeted back to the same set of synapses for local translation. However, a simple experiment demonstrates that the story is a bit more complicated. Our standard paradigm for inducing selective localization involves continuous delivery of HFS so that synapses are active during the time that *Arc* is transported into dendrites. We wondered whether brief HFS would create a signal sufficient to cause selective localization. To test this, we induced LTP by delivering only 20 trains at 400 Hz (a minimal stimulation paradigm for inducing perforant path LTP), and euthanized the rat 2 h later. As illustrated in **Figure 1I**, this minimal stimulation paradigm strongly induced *Arc* mRNA expression as revealed here using non-isotopic *in situ* hybridization, and there was a distinct band of increased labeling in the activated dendritic lamina. Nevertheless, *Arc* mRNA was also transported into distal dendrites. Thus, with the simple LTP paradigm, *Arc* mRNA is concentrated near active synapses, but is also present in non-activated dendritic laminae.

This result has implications in terms of the possible role of Arc in activity-dependent synaptic modification. If Arc protein plays a role in late-phase modifications of synapses that had undergone LTP, this role must not be contingent upon selective targeting to the activated portion of the dendrite. One can argue around this paradox for situations in which Arc is induced by a learning experience. For example, in awake, behaving animals, *Arc* transcription is ongoing at a higher level than in animals that are anesthetized for electrophysiological experiments. Thus, activation of synapses as a result of behavior could generate a docking signal sufficient to capture *Arc* mRNA that is already present in dendrites. This still leaves a missing link, however, between synaptic signals that induce transcription and the signal that mediates docking. The other possible way around the discrepancy is to posit that in learning situations, the patterns of activity that induce *Arc* transcription continue for long enough to refresh the signal for docking so as to allow for capture of *Arc* mRNA transcribed in response to the initial activity that triggered transcription. If this were to work for a one trial learning experience (avoidance conditioning for example) such persistent activity would have to outlast the explicit training experience, however. In either case, the elegant targeting mechanism that can be demonstrated by delivering patterned stimulation to populations of synapses does not quite have the characteristics that would be expected for a consolidation mechanism without postulating some additional processes and mechanisms.

The other implications have to do with mechanisms underlying the selectivity of *Arc* mRNA localization. One interpretation of the finding that *Arc* mRNA was delivered throughout dendrites after LTP was that the signals that mediate docking at active synapses dissipates rapidly so not all newly synthesized *Arc* mRNA is captured in the activated portion of the dendrite unless stimulation is continued. As will be seen below, however, another possible explanation is that the selectivity of localization (presence in active domains and absence in non-active domains) is due to a simultaneous process of activity-dependent *Arc* mRNA degradation.

### TIMING OF Arc PROTEIN SYNTHESIS IN RELATION TO SYNAPTIC CONSOLIDATION

**FIGURE 2:**
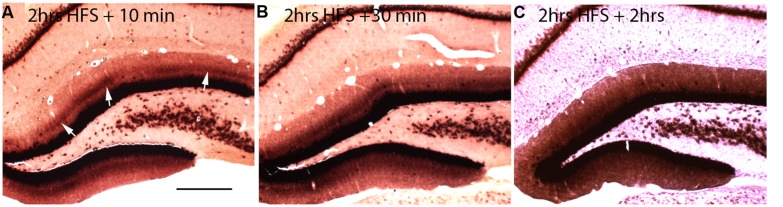
**Changing patterns of *Arc* mRNA localization after discontinuation of synaptic stimulation. (A)** *Arc* mRNA distribution as revealed by NRISH following 2 h of high frequency stimulation of the perforant path to cause localization and 10 min of no stimulation. **(B)** *Arc* mRNA distribution as revealed by NRISH following 2 h of high frequency stimulation of the perforant path to cause localization and 30 min of no stimulation. **(C)** *Arc* mRNA distribution as revealed by NRISH following 2 h of high frequency stimulation of the perforant path to cause localization and 2 h of no stimulation.

If local synthesis of Arc protein is important for making labile changes stable (consolidation), and if the period of consolidation persists for up to an hour after a training experience as behavioral evidence suggests, then *Arc* mRNA should be present at synapses throughout the consolidation period. To determine how long *Arc* mRNA remains localized once it is targeted to synapses, we induced localization by delivering HFS to the MPP for 2 h, which produces a prominent band of *Arc* mRNA in the middle molecular layer of the DG. Stimulation was then discontinued, and rats were killed at various times after the cessation of the stimulation. *in situ* hybridization analyzes revealed that the band of *Arc* mRNA remained distinct 10 and 30 min after the cessation of stimulation, and was still detectable even 2 h after the cessation of HFS (**Figure 2**). By 2 h, however, the band of *Arc* mRNA was less distinct, and levels of labeling for *Arc* mRNA were high throughout the dendritic lamina. At later time points (not shown), *Arc* mRNA was still present in dendrites, but labeling was uniform across the molecular layer rather than being localized in a distinct band in the middle molecular layer.

The change from a discrete band of labeling to a more uniform distribution could be because *Arc* mRNA that is docked near active synapses drifts away when synaptic activation ceases and/or when the docking signal is no longer present. However, it is important to note that like other IEG s, *Arc* mRNA has a short half-life, about 45 min when measured in neurons in culture (Rao et al., 2006). Moreover, the same stimulation conditions that lead to selective localization of *Arc* trigger *Arc* mRNA degradation throughout the molecular layer, which depletes *Arc* mRNA from non-activated portions of the dendrite (Farris et al., 2014). Therefore, it is likely that the mRNA localized near active synapses is continually refreshed with newly synthesized *Arc* mRNA transcribed after the cessation of stimulation. Accordingly, the mRNA that is synthesized after the cessation of stimulation may not get captured in the activated segments once the docking signal dissipates so that newly synthesized *Arc* mRNA is transported throughout dendrites.

As an aside, it should be noted that the continuing transcription of *Arc* after an inducing stimulus is unique to dentate granule cells. Hippocampal pyramidal neurons and presumably other forebrain neurons rapidly shut down *Arc* transcription after an inducing event (Guzowski et al., 1999).

These results indicate that *Arc* mRNA does remain docked near synapses throughout the presumed period of protein synthesis-dependent synaptic consolidation (that is, for 1–2 h after the period of stimulation), and that *Arc* mRNA (and thus protein) is still present in dendrites at high levels for several hours thereafter because of continuing transcription. Is this just leftover, or is Arc protein playing some role even after synaptic changes are stabilized? If elevated levels of Arc protein cause AMPA receptor endocytosis (Chowdhury et al., 2006; Rial Verde et al., 2006), then this would predict that there should be continuing internalization of AMPA receptors throughout the dendritic arbor in the hours after LTP induction. Physiological studies do reveal a slow rundown of LTP, but there have been no reports of slow decreases in synaptic efficacy in *non-stimulated* pathways over the period in which Arc protein levels are elevated after LTP. There can be heterosynaptic depression in inactive synapses, but this occurs immediately after LTP induction and does not develop slowly over the time that Arc protein levels increase.

### HOW DO HIGH LEVELS OF Arc AFFECT SYNAPTIC TRANSMISSION *IN VIVO*?

The findings above raise the question of what increases in Arc protein levels throughout dendrites might mean for synaptic transmission. Several studies now indicate that Arc protein plays a role in facilitating AMPA receptor endocytosis (Rial Verde et al., 2006; Shepherd et al., 2006). For example, over-expression of Arc through transfection of neurons in culture enhances endocytosis of AMPA receptors at synapses resulting in decreases in synaptic strength. These and other data led to the hypothesis that Arc protein is important for reducing synaptic strength in a variety of physiological settings, contributing to synaptic homeostasis (Shepherd et al., 2006). More recent evidence suggests that rapid Arc synthesis is critical for AMPA receptor endocytosis in mGluR dependent LTD (Waung et al., 2008). Importantly, most of the evidence so far comes from studies of neurons *in vitro* or hippocampal slices and use non-physiological means of altering Arc protein levels (for example, transfection, antisense knockdown, or genetic deletion). Accordingly, it was of interest to ask how synaptic transmission *in vivo* would be affected by the dramatic increases in Arc protein levels induced by physiological events.

**FIGURE 3:**
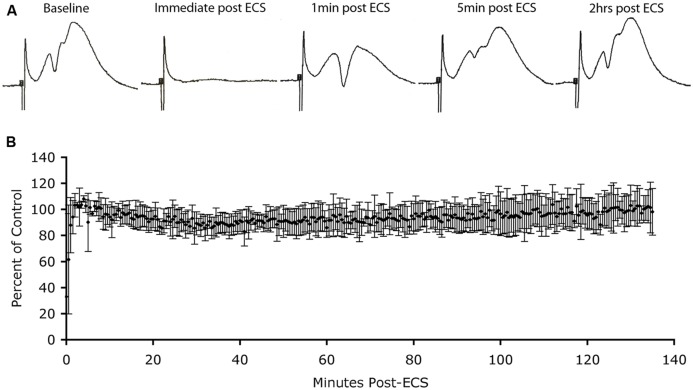
**Absence of depression of synaptic responses as Arc protein levels increase in dendrites. (A)** Perforant path responses before and at various times after ECS. **(B)** Plot of average population excitatory postsynaptic potential (EPSP) slope over time after ECS (*n* = 3 experiments). Note: post-ictal depression immediately after the ECS, rapid recovery of response amplitude and stability of responses for more than 2 h during which Arc protein levels are increasing in dendrites.

To address this question, we again used the model of the perforant path projections to the DG, and the induction of Arc that occurs following ECS. If high levels of Arc trigger AMPA receptor endocytosis, there should be a progressive decrease in synaptic efficacy as Arc protein levels rise after ECS. To test this, we prepared rats (*n* = 3) for acute neurophysiological recording as above, placed stimulating and recording electrodes to record baseline synaptic responses in the perforant path and then delivered ECS to the anesthetized animals. Although the electroconvulsive stimulation did not induce a full tonic–clonic motor seizure due to the anesthesia, there was brief tonic muscle activation and induced seizures in the DG as verified by post-ictal depression of evoked responses lasting 2–3 min (**Figure 3**). Previous studies have confirmed that ECS induced under anesthesia strongly induces *Arc* mRNA (Figure 4 in Steward et al., 1998). Additionally, we have shown previously that Arc protein levels closely mimic *Arc* mRNA levels and distribution in the DG for hours after ECS (Farris et al., 2014). Indeed, our results indicate that in multiple settings, Arc protein distribution mirrors the distribution of *Arc* mRNA.

Immediately after the seizure, we began recording population EPSPs evoked by stimulation of the EC (at one pulse per 30 s). **Figure 3A** illustrates sample responses from one experiment. Post-ictal depression was seen immediately after the ECS, but response amplitude recovered to pre-ECS levels within a few minutes. Thereafter, response amplitude remained stable for the 2 h recording period. In two other animals, response amplitude recovered after the ECS, but not to pre-ECS levels, probably because motor activity during the seizure displaced the recording electrode from the optimal recording site. Our interest was in what happened to response amplitude as Arc protein levels increased over time, so for all cases, extracellular EPSP slopes were normalized to the responses recorded a few minutes after stimulation and the values were expressed as a percent of this control. **Figure 3B** illustrates the average EPSP slope over time post-ECS. Importantly, there was no progressive decrease in EPSP slope during the time that Arc protein levels would be increasing (30–60 min post-ECS). Thus, counter to prediction, substantial increases in Arc protein levels in dentate granule cells as a consequence of seizures did not cause any detectable run down in synaptic efficacy of MPP synapses.

### IS THERE TRANSLATIONAL REPRESSION OF NEWLY SYNTHESIZED *Arc* mRNA?

It is often posited that mRNAs that are delivered to particular intracellular domains are translationally repressed until they reach their destinations. This makes sense if the encoded protein plays a critical role in modifying particular sets of synapses where the mRNA docks. In the case of *Arc*, this is assumed to mean that newly synthesized *Arc* mRNA would be translationally repressed after it moves from the nucleus to the cytoplasm and would remain so until it is delivered to dendrites. Indeed, because *Arc* mRNA is subject to translation-dependent mRNA degradation (see below), *Arc* mRNA would have to be translationally repressed in order to reach synapses on distal dendrites.

To assess whether *Arc* mRNA is translationally repressed as it leaves the nucleus and enters the cytoplasm, we used a behavioral paradigm to induce Arc, and assessed the appearance of newly synthesized Arc protein using immunocytochemistry. The reason for using a behavioral paradigm rather than seizures or synaptic stimulation is the possibility that the high levels of Arc induced by seizures or strong synaptic activation could swamp regulatory mechanisms that would otherwise operate. Accordingly, we used a paradigm in which rats were allowed to explore a novel toy-filled environment. When transferred from a home cage to such an environment, rats explore extensively, investigate the novel objects, and interact with one another. This experience strongly induces *Arc* transcription in neurons throughout the forebrain, and provides an opportunity to assess where within neurons newly synthesized Arc protein first appears.

**FIGURE 4:**
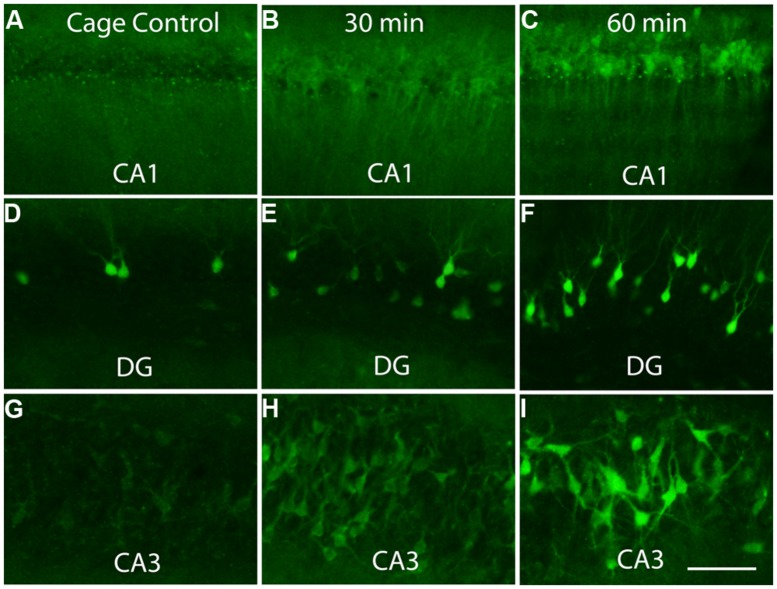
**Appearance of newly synthesized Arc protein after induction by brief exposure to a novel enriched environment.** The panels illustrate immunostaining for Arc protein in the CA1 region of the hippocampus **(A–C)**, dentate gyrus (DG, panels **D–F)** and CA3 region of the hippocampus **(G–I)** in cage controls and 30 and 60 min after exposure to an enriched environment. Note prominent staining of cell bodies at 30 min. Scale bar in I = 100 μm.

Previous studies have revealed that after a discrete training experience, *Arc* transcription can be detected within 2–3 min, and *Arc* mRNA appears in the perinuclear cytoplasm within about 15 min (Guzowski et al., 1999; Miyashita et al., 2009). Here, we assessed Arc protein appearance by immunostaining sections for Arc after different periods of exploration (30 min, 1 and 2 h). Counter to the translational repression hypothesis, abundant amounts of Arc protein are synthesized in the perinuclear cytoplasm (**Figure 4**). Over time, Arc protein then appeared in dendrites with *Arc* mRNA. At all times, however, the highest levels of Arc protein are in the cell body.

One possible explanation for the early appearance of immunostaining in the perinuclear cytoplasm is that translation begins, but is arrested after some portion of the N-terminus of the peptide is synthesized. Then the partial peptide could then be carried out into the dendrites along with the mRNA. This is unlikely to be the explanation, however, because similar results are seen using an antibody generated by Worley and Wu, which is against the C-terminus of Arc (aa 155–396). For this antibody, the appearance of immunostaining reflects completion of synthesis of the C-terminal portion of the peptide.

These results indicate that a substantial proportion of newly synthesized *Arc* mRNA is NOT translationally repressed as it leaves the nucleus, and that substantial amounts of Arc protein are synthesized in the perinuclear cytoplasm. The results do not, however, rule out the possibility that a pool of *Arc* mRNA is translationally repressed so that it can be delivered to distant dendritic sites.

### DISTRIBUTION OF *Arc* mRNA BETWEEN TRANSLATING AND NON-TRANSLATING POOLS

If there is translational repression of *Arc* mRNA, it could occur in two ways—by preventing translation initiation entirely, or by arresting ribosome scanning after initiation. The former model would predict that some fraction of newly synthesized *Arc* mRNA would be in a non-translating pool. To address this question, we assessed the distribution of *Arc* mRNA between translating and non-translating pools using sucrose density gradient analysis.

For this experiment, mice were pretreated with scopolamine (1 mg/kg IP) and were killed 30 min later by exposure to isoflurane and decapitation. This was done as a control for other experiments in which the distribution of *Arc* mRNA was evaluated after seizures (to be reported elsewhere). The cortex was homogenized and the post-nuclear supernatant was run on 20–50% sucrose gradients. Fractions were collected and *Arc* mRNA levels were assayed by quantitative PCR and normalized based on levels of luciferase mRNA added as an internal standard.

**FIGURE 5:**
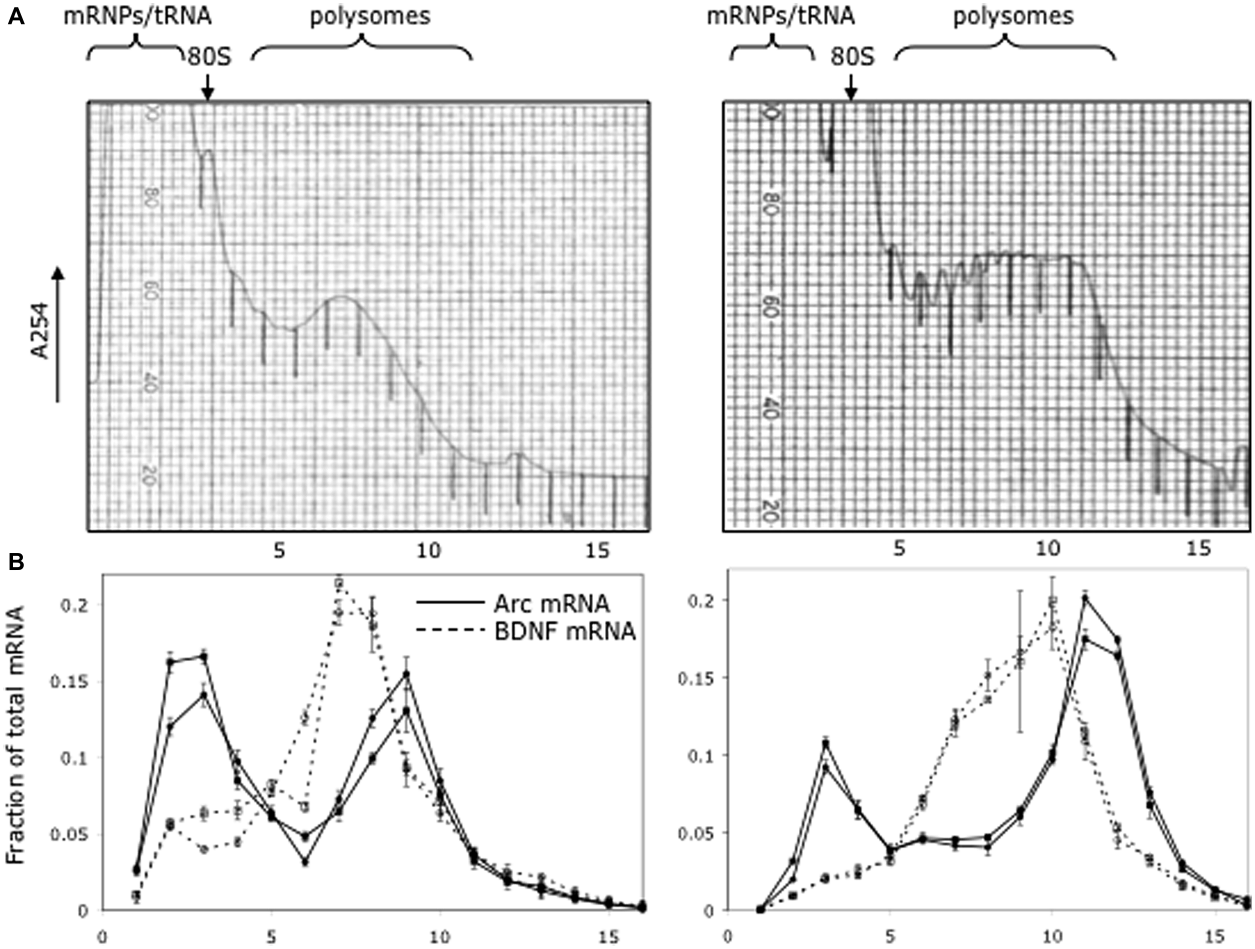
**Distribution of *Arc* and *Bdnf* mRNAs on polyribosome gradients. (A)** Post-mitochondrial adult mouse brain lysate from two littermates was spun on 20–50% sucrose gradients to separate mRNP, monosome and polysome fractions. 0.72 ml fractions were collected with UV monitoring of RNA levels at A254. Fraction numbers are indicated below the panel. **(B)** Quantitative RT-PCR was used to measure *Arc* (solid lines) and *BDNF* (dotted lines) mRNA level in each fraction as described in Methods. Data are plotted as a fraction of the total recovered from the gradient, and are normalized for RNA recovery from each fraction. Two biologic replicates are individually shown on the graph. Error bars reflect standard deviation of three technical replicates.

As illustrated in **Figure 5**, which illustrates the results from two different mice, a substantial proportion of *Arc* mRNA was present in the non-translating pool (gradient fractions 2–3). In addition, there was a second peak in *Arc* mRNA levels in the slowly migrating end of the polysome fraction (fractions 8–10). The fact that the peak in *Arc* mRNA is in fractions 8–10 suggests loading of multiple ribosomes. Importantly, the distribution of *Arc* mRNA was strikingly different than BDNF mRNA, which was concentrated in the polysome fraction. These results are consistent with the hypothesis that a substantial portion of the total *Arc* mRNA in cortical neurons *in vivo* is in a pool that is not being translated and may be translationally repressed.

### ACTIVITY-DEPENDENT *Arc* mRNA DEGRADATION

A key recent discovery was that *Arc* mRNA is subject to a previously unknown process of synaptically driven mRNA degradation that contributes to the selectivity of *Arc* mRNA localization (Farris et al., 2014). This discovery came from studies that tested whether *Arc* mRNA that was already in dendrites would re-localize to active synapses. For this, we used the ECS-Perforant Path stimulation paradigm in which *Arc* is induced by an ECS, time is allowed for the mRNA to move throughout dendrites, then HFS synaptic is delivered, which causes the mRNA to localize in the activated dendritic lamina (Steward and Worley, 2001b). *Arc* mRNA begins to accumulate in the activated dendritic lamina within 15 min after stimulation begins. At the same time, *Arc* mRNA is depleted from non-activated portions of the dendrite, resulting in a strikingly selective pattern of localization in which there are high levels of *Arc* mRNA in the activated lamina and virtually none in the non-activated lamina.

Three mechanisms could explain the accumulation of *Arc* mRNA in active lamina and depletion from inactive laminae: (1) *Arc* mRNA already in dendrites could re-localize from inactive to active dendritic domains; (2) newly transcribed *Arc* mRNA that is in transit might dock at active synapses while existing mRNA is degraded; (3) the selective pattern of localization could be due to mRNA degradation in inactive domains and stabilization near active synapses.

To distinguish between these three mechanisms, we induced *Arc* with an ECS, waited until *Arc* mRNA was transported throughout dendrites, infused actinomycin D (Act-D) to block further transcription, and then initiated synaptic stimulation. If *Arc* mRNA already in dendrites re-localizes to active synapses (mechanism 1) this should occur even if transcription is blocked. We found, however, that when *Arc* transcription was blocked by Act-D, there was no accumulation of *Arc* mRNA near active synapses, and virtually all *Arc* mRNA already in dendrites was rapidly degraded. The rapid degradation of *Arc* mRNA throughout dendrites in the presence of Act-D also eliminated mechanism #3; there is clearly no stabilization of existing *Arc* mRNA near active synapses. Thus, it is only newly transcribed *Arc* mRNA that localizes at active synapses and *Arc* mRNA already in dendrites is rapidly degraded in an activity-dependent fashion (Farris et al., 2014).

### ACTIVITY-DEPENDENT DEPLETION OF *Arc* mRNA REQUIRES ONGOING PROTEIN SYNTHESIS

A possible mechanism for the mRNA depletion is that *Arc* mRNA is subject to nonsense-mediated mRNA decay (NMD). *Arc* mRNA is a canonical candidate for NMD because of the presence of splice junction sites in the 3′UTR downstream of the stop codon (Giorgi et al., 2007). Proteins of the exon junction complex (EJC) are required for splicing in the nucleus and remain bound to *Arc* mRNA as it moves into the cytoplasm. EJC’s downstream of a stop codon are the canonical signal for triggering NMD when a translating ribosome reaches the stop codon. If activity-dependent degradation occurred via NMD, it would NOT occur when protein synthesis is blocked. Consistent with this prediction, local infusion of protein synthesis inhibitors into the DG of animals that received ECS and subsequent perforant path stimulation completely prevented activity-dependent depletion of *Arc* mRNA that would otherwise occur. For example, with infusion of CHX 1.5 h post-ECS but prior to HFS of the MPP, *Arc* mRNA is dramatically increased over the region of recently activated synapses as well as throughout the dendrite. When CHX is locally infused into the DG 1.5 h post-ECS and animals are sacrificed 1.5 h later, levels of *Arc* mRNA are moderately increased in the dendrites of granule cells (Farris et al., 2014). These data demonstrate that *Arc* mRNA in dendrites is subject to translation-dependent degradation AND that this degradation is dramatically enhanced by synaptic activity (Farris et al., 2014).

The data above on ribosome loading are pertinent in terms of understanding how many Arc protein molecules could be synthesized by a particular *Arc* mRNA molecule before it is degraded. In the extreme, translation-dependent degradation could destroy the mRNA after a single Arc protein molecule was synthesized. On the other hand, if mRNA degradation begins at the 5′ end due to de-capping, and if translation could continue via ribosomes already loaded onto the mRNA, this would mean that multiple protein molecules could be synthesized before the mRNA was degraded. This still means that overall synthesis of Arc protein would be tightly controlled, but not necessarily on a single molecule basis.

### SOME PUZZLING DATA ON mRNA BINDING PROTEINS

If *Arc* mRNA is in fact regulated by NMD, then the protein components of the EJC must be carried into dendrites along with *Arc* mRNA. In keeping with this hypothesis, Giorgi et al. (2007) showed significant co-localization of *Arc* mRNA and the key EJC factor eIF4AIII in the dendrites of neurons in culture. Another prediction, however, is that when *Arc* mRNA is induced by physiological events *in vivo*, levels of EJC proteins should increase in dendrites in proportion to increases in levels of *Arc* mRNA. This question was addressed in Giorgi et al. (2007) but the results were negative; there were no detectable increases in eIF4AIII protein levels in dendrites of dentate granule cells following ECSs when *Arc* mRNA levels are strikingly increased (results were presented in a supplementary figure). Giorgi et al. (2007) argue that this is actually the expected result for two reasons: *Arc* is only one of several dendritic mRNAs that have associated EJC proteins (the tested example being 4AIII), and *Arc* represents a small fraction of the total dendritic mRNA. The first assertion may be true, but there are no data to support the latter. Only 4 mRNAs have been shown to be abundant in dendrites of forebrain neurons by *in situ* hybridization; the mRNAs for *αCaMKII, Map2, Dendrin*, and *Arc* when it is induced. Although *in situ* hybridization does not provide a definitive measure of mRNA copy number or molar levels, it does seem likely that when induced by seizures, *Arc* mRNA rises to a level that is more than a small fraction of the total dendritic mRNA. Thus, it remains surprising to us that there are no detectable increases in levels of EJC components in dendrites when *Arc* mRNA levels are strongly induced.

**FIGURE 6:**
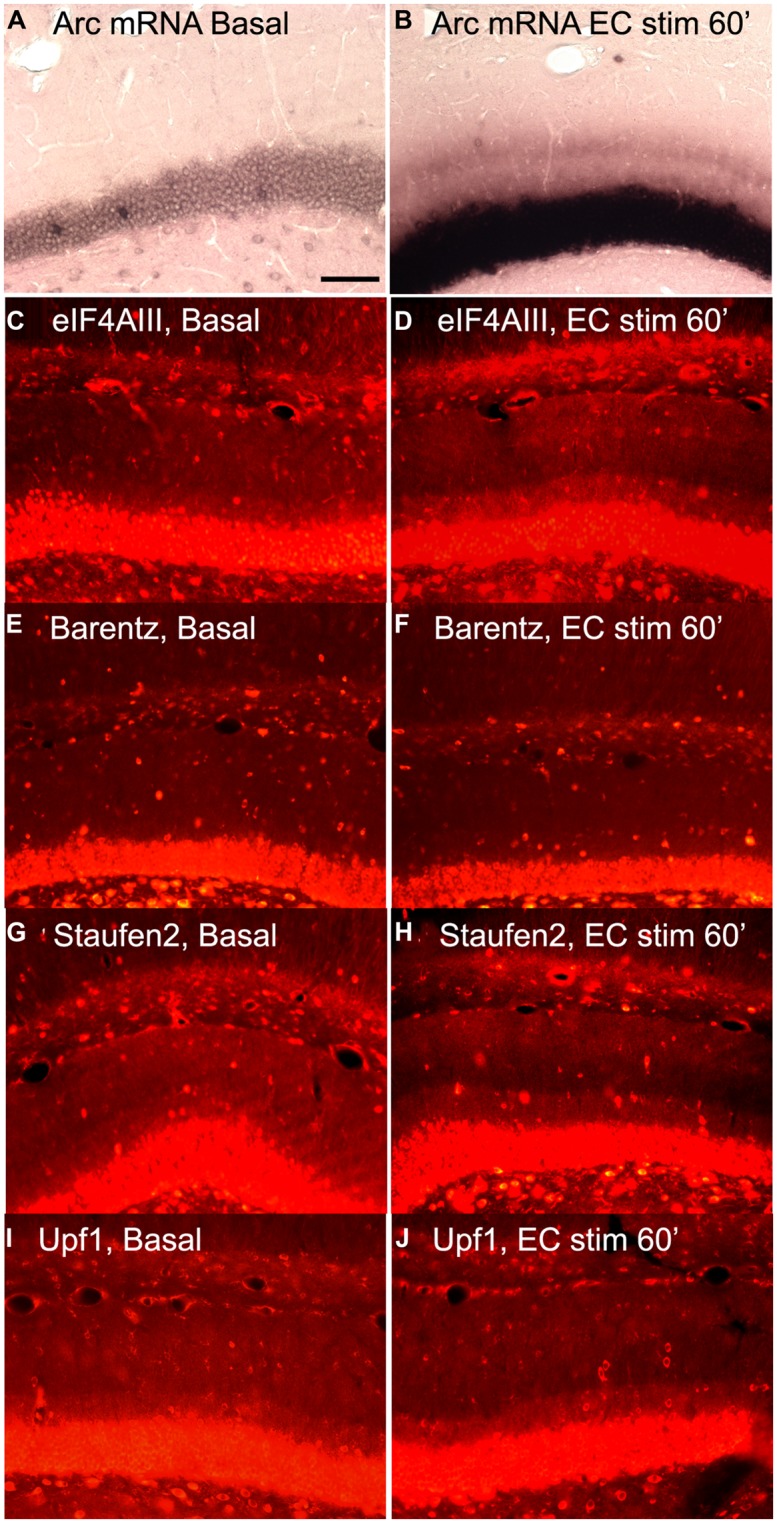
**Distribution of NMD proteins after synaptic stimulation. (A,C,E,G,I)** Basal levels of *Arc* mRNA, eIF4AIII, Barentz, Staufen2, and Upf1expression on the contralateral side to stimulation, respectively. **(B,D,F,H,J)** *Arc* mRNA, eIF4AIII, Barentz, Staufen2, and Upf distribution after 1 h of synaptic stimulation, respectively. Scale bar is 100 μm.

Based on this logic, we re-assessed the question of whether increases in *Arc* mRNA in dendrites were associated with increases in proteins involved in NMD. We induced *Arc* transcription and synaptic targeting using our standard protocol involving HFS of the perforant path. **Figure 6A** illustrates basal levels of Arc mRNA; **Figure 6B** illustrates Arc mRNA distribution after 60 min HFS. Rats received HFS for 1 h, and were then prepared for *in situ* hybridization and immunostaining for eIF4AIII. As expected, *Arc* mRNA was strongly induced and targeted to the activated dendritic laminae. Immunostaining of nearby sections for 4AIII revealed no increase in 4AIII levels in the dendritic laminae in comparison to the non-stimulated control side (**Figures 6C,D**). Surprisingly, there was a small but consistent decrease in 4AIII immunolabeling in the activated lamina in which *Arc* mRNA accumulates (**Figure 6D**). Importantly, stimulation did not lead to the depletion of all RBPs tested. In fact, Barentsz, another core EJC protein showed no significant change in immunostaining in the same tissue (**Figures 6E,F**).

We further reasoned that if translation-dependent degradation of *Arc* mRNA occurs throughout dendrites, the enzymes recruited to mRNAs targeted for NMD must be present constitutively in the dendritic laminae or perhaps accompany mRNAs as they are transported into dendrites. To address this, sections from experiments described above were immunostained for Upf1 and Staufen2, which have been shown to be necessary for certain forms of NMD. In control tissue, immunostaining revealed that Upf1 and Staufen2 were present at high levels in neuronal cell bodies, but also present throughout dendritic laminae (**Figures 6G,I**). However, stimulation did not lead to an increase in immunostaining for either Upf1 or Staufen2. In fact, as with eIF4AIII, there was a consistent decrease in the activated lamina, leading to a tri-laminar pattern of staining (**Figures 6H,J**). The reasons for the depletion of Upf1, Staufen2, and eIF4AIII in the activated lamina are not clear, but could indicate that these RBPs dissociate from *Arc* once docked or that they are degraded in conjunction with the degradation of *Arc* mRNA.

These data do not, however, definitely rule out the possibility that these RBPs accompany *Arc* mRNA into dendrites. It may be that there are high levels of RBPs constitutively that are not associated with dendritic mRNAs. In this way, NMD proteins would be available in excess, and would not be rate limiting for mRNA degradation. Additionally, there are likely 100s, possibly 1000s, of mRNAs localizing in different regions of the dendrite so that we might not have the spatial resolution to detect even large-scale changes in RBPs as seen with *Arc*. Immunostaining in the dendritic layer may also reflect the presence of RBPs in glial cells and their processes. In this case, increases in NMD proteins consequent to *Arc* mRNA delivery might not cause detectable increases in overall NMD protein levels.

## DISCUSSION

Scientific reviews often focus on data that fit together into a consistent story, and perhaps omit data that donot make sense in the context of the story. Our goal here was to take the opposite approach of focusing on data that do not yet fit in. In our view, these paradoxes demonstrate that a comprehensive understanding of the role of Arc in synaptic modification and memory has not yet been achieved. By laying out the paradoxes, we hope to stimulate creative thought and motivate further experiments that will clarify the issues and allow a reformulation of hypotheses. The key paradoxes are:

(1) After induction of LTP, *Arc* mRNA accumulates to some degree at active synapses, but is also present throughout the rest of the dendrite. This implies that in this model of synaptic plasticity, whatever role Arc plays does not require selective targeting to the synapses being modified.
(2) Strong induction of synthesis of Arc protein does not cause a rundown of synaptic potency in dentate granule cells *in vivo*. This is in contrast to studies of the effects of transfection-mediated over-expression of Arc in CA1 pyramidal neurons *in vivo*. One possible explanation of the disparity is that exogenous over-expression does not actually reveal Arc’s role in physiological settings *in vivo*.
(3) A substantial pool of *Arc* mRNA is not associated with translating ribosomes. Nevertheless, after induction, substantial amounts of Arc protein are synthesized in the perinuclear cytoplasm. Thus, at least some newly transcribed *Arc* mRNA is not translationally repressed. This may mean that of *Arc* mRNA is present in different pools in the cytoplasm.
(4) Immunocytochemical analyzes indicate that there are no detectable increases in levels of EJC proteins in dendrites in conjunction with increases in levels of *Arc* mRNA. This may mean that EJC proteins do not accompany *Arc* mRNA into dendrites that EJC proteins are rapidly degraded, or that EJC proteins rapidly shuttle back to the nucleus.

There are of course caveats for all of these conclusions, and it is clear that there is still much to be learned about the mechanisms underlying *Arc* transport and localization and the role of Arc at synapses. Some of the caveats and un-resolved questions are discussed further below.

### SELECTIVITY OF Arc LOCALIZATION

Our previous studies document that *Arc* mRNA localizes selectively near active synapses when brief trains of HFS are delivered during the time that *Arc* mRNA is transported into dendrites. Here, we show that *Arc* mRNA is present throughout dendrites following the induction of LTP. One possible explanation is that the signal that causes *Arc* mRNA to dock at active synapses is short-lived so that the signal dissipates by the time *Arc* mRNA reaches the active lamina. However, this does not accord with the fact that once localization is induced by prolonged stimulation, newly synthesized *Arc* continues to localize selectively for a considerable period of time after HFS is discontinued. Clearly, there is more to learn about how the signal for targeting is generated and maintained.

Whether and how these mechanisms operate in physiological settings remains to be determined. Repeated delivery of high frequency trains of stimuli seems non-physiological *a priori*, but neurons do fire in high frequency bursts and it is possible that after a learning event, neurons continue to fire periodically, leading to continual refreshment of the mRNA localization signal at post-synaptic sites on the recipient neurons. Thus, the patterns of HFS delivered here are not necessarily out of the physiological range except that large numbers of synapses are being activated simultaneously.

The second issue is whether selective localization of *Arc* mRNA and protein in the activated portion of dendrites indicates localization at individual synapses. Our studies on the distribution of *Arc*/MS2 mRNA indicate precise localization at the base of dendritic spines, but it is not known whether Arc protein localizes with similar precision. Another unknown is whether *Arc* mRNA and protein localize selectively at synapses that have undergone potentiation, depression or both. In this regard, one recent study demonstrates that an Arc/GFP fusion protein localizes selectively at inactive rather than active synapses (Okuno et al., 2012), consistent with the proposed role for Arc in homeostatic plasticity. Also, both our studies and those of Okuno et al. (2012) assess localization of fusion transcripts or fusion proteins in neurons in culture. In our view, what is really needed is an assessment of these questions in neurons *in vivo* when Arc is induced physiologically in learning situations. However, this will be technically difficult because it will require ways to identify potentiated vs. depressed synapses after a learning paradigm and simultaneously assess the distribution of *Arc* mRNA and protein at a synapse-by-synapse level.

### HIGH LEVELS OF Arc PROTEIN DO NOT INVARIABLY LEAD TO DECREASES IN SYNAPTIC EFFICACY *IN VIVO*

Current hypotheses about how Arc protein mediates receptor endocytosis and LTD are based largely on studies involving over-expression of Arc in neurons in culture (for example Rial Verde et al., 2006). Our findings here reveal that strong induction of *Arc* mRNA and protein expression by a seizure does not lead to any detectable run-down of synaptic efficacy as Arc protein levels rise in dendrites. One possible explanation for the discrepant findings is that previous studies involving exogenous expression of Arc in neurons *in vitro* do not reveal mechanisms that operate when Arc is induced by physiological events *in vivo*. Alternatively, it is possible that in our paradigm, the seizure itself could modify or block mechanisms in dentate granule cells that would otherwise operate, such as those required for AMPAR endocytosis. This would mean that the functions of Arc at the synapse depend on other processes, which would certainly not be surprising. Determining whether and how Arc function varies depending on other circumstances at synapses will be critical to our understanding of Arc function.

### SYNTHESIS OF Arc IN NEURONAL CELL BODIES

Our findings here reveal that substantial amounts of Arc protein are synthesized in neuronal cell bodies very shortly after *Arc* transcription is induced. These results, together with the results showing that a substantial portion of *Arc* mRNA is associated with translating ribosomes, indicates that at least some newly synthesized *Arc* mRNA is not translationally repressed. This is important because some models of mRNA transport suggest the importance of translational repression until the mRNA reaches its final destination, especially for mRNAs like *Arc* that are subject to translation-dependent mRNA decay (Bramham et al., 2010). On the other hand, it is important to recall that Arc protein also accumulates in the nucleus where it may have a different role than it does at synapses (Korb et al., 2013). Thus, there may be two (or perhaps more) pools of *Arc* mRNA and the protein made by transcripts in the different pools has different functions.

### *Arc* mRNA BINDING PROTEINS

Current models of mRNA transport propose that RBPs play a critical role in mRNA trafficking (Bagni and Greenough, 2005; Bramham et al., 2010). Most models posit that some RBPs bind nascent transcripts in the nucleus; examples include the EJC proteins that are thought to accompany the mRNA into the cytoplasm (Giorgi et al., 2007; Doyle and Kiebler, 2011). Other RBPs associate with the mRNA in the cytoplasm and target it to the transport machinery. Our immunocytochemical experiments here represent a preliminary test of one aspect of this hypothesis, and indicate that increases in *Arc* mRNA in dendrites are not accompanied by increases in the levels of candidate RBPs in dendrites. Clearly these findings are preliminary, and there are many possible explanations. Nevertheless, our results indicate that the situation is more complex than simple models might predict. Combining paradigms that physiologically induce *Arc* with techniques that allow for precise identification of associated RBPs may reveal where and how EJC proteins and other RBPs function in *Arc* trafficking and translation.

To highlight some of the inconsistencies, it is useful to consider our data here in terms of a few key issues summarized in **Table 1** regarding delivery of *Arc* mRNA into dendrites, selective localization of *Arc* mRNA and protein at synapses, local synthesis of Arc protein in dendrites, and role of dendritically synthesized Arc at synapses. The table does not include studies documenting aspects of induction of *Arc* transcription during learning experiences, behavior-specific induction of *Arc* in neural networks, functions of *Arc* in other cellular domains (for example the nucleus) and the growing literature on localization and local translation of other mRNAs in dendrites.

**Table 1:** Key findings related to delivery of *Arc* mRNA to dendrites, selective localization of *Arc* mRNA at synapses, local synthesis of Arc protein in dendrites, and role of dendritically synthesized Arc at synapses.

Discovery	Reference	Notes
Discovery of *Arc/Arg3.1*, an immediate early gene (IEG) that is not a transcription factor. *Arc*/*Arg3.1* mRNA is delivered throughout dendrites.	Link et al. (1995); Lyford et al. (1995)	Identified *Arc* through screens for genes whose expression was induced by neuronal activity and learning experiences. Lyford et al. (1995) also provided evidence that *Arc* was an effector gene and not a transcription factor.
*Arc* mRNA is targeted to dendrites based on a sequence in the RNA.	Wallace et al. (1998)	*Arc* mRNA is delivered to dendrites when protein synthesis is blocked, indicating that the mRNA itself contains signals sufficient for dendritic delivery.
*Arc* mRNA localizes selectively in dendritic domains contacted by active synapses.	Steward et al. (1998)	Repeated delivery of short trains of high frequency stimulation to the perforant path caused *Arc* mRNA to localize selectively in activated dendritic domains.
Knockdown of *Arc* mRNA with antisense oligonucleotides impairs late-phase hippocampal LTP and memory consolidation.	Guzowski et al. (2000)	Intra-hippocampal infusions of *Arc* antisense oligonucleotides impairs the maintenance phase of LTP without affecting induction and impairs long-term memory but not acquisition in a spatial water maze.
Localization of *Arc* mRNA near active synapses depends on NMDA receptor activation.	Steward and Worley (2001b)	Intra-hippocampal infusion of NMDA receptor antagonists during high frequency perforant path stimulation prevented activity-dependent targeting of *Arc* mRNA to active dendritic domains.
Arc knockout mice have impaired long term memory and deficits in late-phase LTP and LTD.	Plath et al. (2006)	Phenotypic characterization of Arc knockout mice using different memory assessment tasks and assessing both hippocampal and perforant path LTP and LTD.
Arc protein interacts with endophilin and dynamin to enhance AMPA receptor endocytosis at synapses.	Chowdhury et al. (2006)	Biochemical studies document interaction between Arc protein and specific isoforms of endophilin. Over-expression of Arc/EGFP fusion protein in neurons in culture reduces numbers of surface GluR1 receptors.
Over-expression of Arc/EGFP fusion proteins reduce AMPA receptor-mediated synaptic transmission.	Rial Verde et al. (2006)	Neurons in hippocampal slices transfected to over-express Arc/EGFP have lower amplitude AMPA-receptor-mediated MESCs. This effect is prevented by RNAi knockdown of Arc, or by deleting a region of Arc that interacts with endophilin 3.
High levels of Arc prevent homeostatic rescaling of AMPA-receptors in neurons in culture after chronic blockade of neuronal activity.	Shepherd et al. (2006)	In an established model of homeostatic rescaling in neurons in culture, over-expression of Arc/EGFP prevents increases in AMPA receptors that otherwise occur with chronic activity blockade. Arc knockdown leads to increases in basal surface levels, and occludes homeostatic rescaling.
Selective localization of *Arc* mRNA near active synapses requires actin polymerization and MAP kinase activation.	Huang et al. (2007)	Local infusion of inhibitors of actin polymerization or MAP kinase during high frequency perforant path stimulation prevented the targeting of *Arc* mRNA to active dendritic domains.
Knockdown of *Arc* mRNA with antisense oligonucleotides results in rapid reversal of perforant path LTP and disruption of actin polymerization.	Messaoudi et al. (2007)	Delivery of antisense oligonucleutides into the hippocampus 2 h after induction of perforant path LTP led to rapid reversal of LTP and disrupted the band of polymerized actin triggered by LTP induction.
Fusion transcripts containing the 3′UTR of *Arc* mRNA are transported bi-directionally in dendrites at rates up to 70 μm/min.	Dynes and Steward (2007)	Neurons in culture were transfected with fusion transcripts that include the 3′UTR of *Arc* along with a sequence that binds the bacterial protein MS2 together with an MS2/GFP fusion protein.
*Arc* mRNA is subject to nonsense-mediated decay (NMD).	Giorgi et al. (2007)	Noted that *Arc* mRNA has a splice site in the 3′UTR, that splice junction complex (SJC) proteins accompany *Arc* mRNA into the cytoplasm and that *Arc* mRNA degradation is blocked by inhibiting protein synthesis.
Induction of LTD via activation of mGluRs induces *Arc* synthesis via phosphorylation of eEF2.	Park et al. (2008)	Both paired pulse and mGluR dependent LTD are impaired in Arc KO mice. mGluR-dependent activation of *Arc* translation is disrupted in mice lacking Fragile X mental retardation protein (Fmr1 knockout mice).
Induction of mGluR-dependent LTD triggers Arc protein synthesis in dendrites and increased endocytosis of AMPAR; are not seen with NMDAR-dependent LTD.	Waung et al. (2008)	LTD was induced by DHPG stimulation, measuring Arc expression in neurons in culture and dendrites in hippocampal slices. Surface AMPA receptors were assessed in neurons in culture. Knockdown of Arc with antisense oligonucleotides in hippocampal slices blocks L-LTD. Increases in Arc protein.
Fusion transcripts containing the 3′UTR of *Arc* mRNA localize selectively at the base of dendritic spines.	Dynes and Steward (2012)	Stationary Arc/MS2 fusion transcripts in neurons in culture are precisely localized in a small microdomain at the base of dendritic spines.
Arc/EGFP fusion protein accumulates preferentially at inactivated synapses that had previously been active. Arc accumulation at spines is impaired by knockdown of CaMKIIß.	Okuno et al. (2012)	Assessed homeostatic plasticity in neurons in culture using a strategy to silence presynaptic release at some synapses. Showed that Arc/GFP fusion protein accumulates preferentially at synapses that had previously been active but were silent.
Experience-induced Arc primes hippocampal neurons for subsequent induction of mGluR-dependent LTD.	Jakkamsetti et al. (2013)	Induction of Arc in hippocampal neurons as a result of exposure to a novel environment does not in and of itself lead to decreases in synaptic efficacy but does prime neurons for subsequent induction of mGluR-dependent LTD. Multiple exposures to a novel environment do lead to LTD.
*Arc* mRNA is degraded in dendrites in response to synaptic activity.	Farris et al. (2014)	Repeated high frequency activation of the perforant path enhanced degradation of *Arc* mRNA that is already present in dendrites, which contributes to the selectivity of localization near active synapses. Demonstrated that it is primarily newly transcribed *Arc* mRNA that localizes in activated dendritic domains.

In terms of delivery, we are unaware of findings that counter the conclusion that *Arc* mRNA is delivered into dendrites based on a sequence in the mRNA itself, likely within the 3′UTR. Our studies here of RBPs donot provide further evidence supporting the conclusion that proteins involved in splicing accompany *Arc* mRNA into dendrites (Giorgi et al., 2007), but our data are also not strong evidence against the idea.

In terms of localization at synapses, studies of native *Arc* mRNA or exogenously expressed constructs containing the 3′UTR of *Arc* indicate selective targeting of the mRNA to dendritic domains contacted by active synapses (Steward et al., 1998; Steward and Worley, 2001b), and that this localization depends on activation of NMDA receptors and downstream effectors (Huang et al., 2007). In contrast, one study suggests that Arc/EGFP fusion protein is preferentially targeted to inactive synapses (Okuno et al., 2012).

In terms of the role of Arc in modulating synaptic efficacy by regulating AMPA receptor trafficking, studies involving exogenous over-expression of Arc/EGFP are consistent in reporting enhanced AMPA receptor endocytosis leading to decreased synaptic efficacy. In contrast, two studies (here, and Jakkamsetti et al., 2013) report that physiological induction of native Arc *in vivo* in and of itself does not lead to a decrease in synaptic efficacy as Arc levels increase in dendrites. It is noteworthy that Jakkamsetti et al. (2013) report that physiological induction of Arc does prime neurons for LTD.

There is a lack of clarity about Arc’s role in LTP. Intra-hippocampal injections of *Arc* antisense oligonucleotides lead to failure to maintain late phase LTP (Guzowski et al., 2000) and lead to rapid reversal of established LTP (Messaoudi et al., 2007). The LTP phenotype in Arc knockout mice is more complicated (Plath et al., 2006). Most importantly, to our knowledge, there is no compelling evidence to explain how Arc would operate in both LTP and LTD.

## Conflict of Interest Statement

The authors declare that the research was conducted in the absence of any commercial or financial relationships that could be construed as a potential conflict of interest.
